# Feasibility, safety and tolerability of accelerated dobutamine stress echocardiography

**DOI:** 10.1186/1476-7120-5-40

**Published:** 2007-11-21

**Authors:** Giovanni Minardi, Carla Manzara, Giovanni Pulignano, Paolo G Pino, Herribert Pavaci, Martina Sordi

**Affiliations:** 1 Cardiodiagnostica non invasiva, Department of Cardiology and Cardiovascular Surgery, Azienda Ospedaliera San Camillo-Forlanini, Rome, Italy; 2 Second Division of Cardiology, Department of Heart and Great Vessels Attilio Reale, "Sapienza", University of Rome, Italy

## Abstract

A continuous infusion of a single high dose of dobutamine has been, recently, suggested as a simple and effective protocol of stress echocardiography. The present study assesses the feasibility, safety, and tolerability of an accelerated dobutamine stress protocol performed in patients with suspected or known coronary artery disease. Two hundred sixty five consecutive patients underwent accelerated dobutamine stress echocardiography: the dobutamine was administered at a constant dose of 50 μg/kg/min for up to 10 minutes. The mean weight-adjusted cumulative dose of dobutamine used was 330 ± 105.24 μg/kg. Total duration of dobutamine infusion was 6.6 ± 2.1 min. Heart rate rose from 69.9 ± 12.1 to 123.1 ± 22.1 beats/min at peak with a concomitant change in systolic blood pressure (127.6 ± 18.1 vs. 167.6 ± 45.0 mmHg). Dobutamine administration produced a rapid increase in heart rate (9.4 ± 5.9 beats/min^2^). The side effects were similar to those described with the standard protocol; the most common were frequent premature ventricular complexes (21.5%), frequent premature atrial complexes (1.5%) and non sustained ventricular tachycardia (1.5%); among non cardiac symptoms the most frequent were nausea (3.4%), headache (1.1%) and symptomatic hypotension (1.1%). No major side effects were observed during the test. Our data demonstrate that a continous infusion of a single high dose of dobutamine is a safe and well tolerated method of performing stress echocardiography in patients with suspected or known coronary artery disease. This new protocol requires the administration of lower cumulative dobutamine dose than standard protocol and results in a significant reduction in test time.

## Background

Dobutamine stress echocardiography (DSE) is commonly used to assess the extent, location, and severity of coronary artery disease (CAD) and myocardial viability [1-6]. The duration and infusion dose of dobutamine for the assessment of myocardial ischemia and viability has been studied extensively [7-10]. Currently, in patients with suspected or known CAD, most laboratories use stepwise increments of dobutamine at 3-minute intervals, which has evolved from the commonly used exercise treadmill protocols. However, steady-state dobutamine levels during dobutamine infusion are not obtained for up to 10 minutes [5,7,11]. Consequently, the full effect of any infusion rate of dobutamine is not obtained before the dobutamine dose has advanced to the next level [12] and plasma dobutamine concentrations increase rapidly and non-linearly during the test [1,13,14]. Furthermore, previous studies have shown that patients treated with beta-adrenergic antagonists frequently fail to reach target heart rate [15,16]. In these patients, the addition of atropine has been reported to improve the sensitivity of DSE by increasing the heart rate response [17]. Therefore, a continuous infusion of a single high dose of dobutamine has been suggested as a simple and effective protocol of DSE [1,18]. The present study assesses the feasibility, safety, and tolerability of an accelerated dobutamine stress protocol (ADSE) performed in patients with suspected or known CAD.

## Methods

### Patient Population

Between March 2002 and October 2007, at San Camillo Hospital, we prospectively enrolled 265 consecutive patients (mean age 63.3 ± 11.6; males 185). All patients underwent ADSE. Demographic and clinical characteristics of patients are reported in Table 1.

**Table 1:** Clinical characteristics of 265 patients

*Clinical parameters*	*n*	*%*
Age (yrs)	63.3	± 11.6
Male sex	185	69.8
*History*		
Hypertension	142	53.6
Diabetes mellitus	51	19.2
Dyslipidemia	116	43.8
Cigarette smoker	54	20.4
Previous MI	185	69.8
Previous CABG	19	7.2
Previous PTCA	112	42.3
*Medication*		
β-blockers	77	29.1
Calcium channel blockers	72	27.2
Nitrates	58	21.9
Other	247	93.2

Values are expressed as mean ± SD or number (%) of patients unless otherwise stated.

CABG, coronary artery bypass grafting; PTCA, percutaneous transluminal coronary angioplasty;MI, myocardial infarction.

Indications for ADSE included evaluation of angina (n = 55), atypical chest pain (n = 22), ECG uninterpretable ECG (n = 15), chest pain in hypertensive patients (n = 11), risk stratification after myocardial infarction (n = 180), follow-up of PTCA (percutaneous transluminal coronary angioplasty) (n = 113) or CABG (coronary artery bypass grafting) (n = 20), preoperative risk assessment in non cardiac surgery (n = 3) and miscellaneous reasons (n = 16).

Patients were on therapy, if indicated, including a drug combination of β-blockers, long-acting nitrates, calcium antagonists, others (diuretics, aspirin, statins, ace inhibitors). (Table 1).

Whenever possible or indicated, beta-adrenergic antagonists were withheld for at least 72 h before stress echocardiography. Informed consent was obtained from all patients.

### Dobutamine Accelerated infusion protocol

Dobutamine was administered at a constant dose of 50 μg/kg/min for up to 10 minutes. All dobutamine stress tests were performed under continuous 12-lead electrocardiographic (ECG) and non-invasive blood pressure monitoring. After obtaining rest heart rate, blood pressure and left ventricular two-dimensional echocardiographic images, the dobutamine infusion was initiated. Diagnostic endpoints of the test were:

positive echocardiogram(new onset wall motion abnormalities or worsening of baseline dissinergy); achievement of 85% of maximal predicted heart rate (220 - age); severe chest pain and/or diagnostic ST-segment changes.

The test was stopped without diagnostic endpoints for: Intolerable symptoms; hypertension(systolic blood pressure > 220 mmHg, diastolic blood pressure > 120 mmHg); hypotension (> 30 mmHg fall of blood pressure); supraventricular arrhythmias (supraventricular tachycardia or atrial fibrillation); or ventricular arrhythmias (ventricular tachycardia; frequent, polymorphous, premature ventricular beats). Dobutamine infusion was discontinued after 10 minutes or for 1 of the end points used in the standard protocol.

### Echocardiographic analysis

Echocardiographic images were acquired at rest and during stress and recovery. Echocardiograms were recorded on videotapes and were also digitized on optical disk and displayed side by side in quad-screen format to facilitate comparison of images. The left ventricular wall was divided into 16 segments and scored using a 4-point scale, where 1 = normal, 2 = hypokinesia, 3 = akinesia, and 4 = dyskinesia [19]. Wall motion score index was derived by dividing the sum of individual score of the 16 segments by 16. The interpretation of images was performed by 2 experienced observers without knowledge of the patients' clinical data. In case of disagreement, a majority decision was achieved by a third observer. Ischemia was defined as new or worsening wall motion abnormalities.

### Electrocardiographic analysis

Electrocardiographic changes were considered to be ischemic if an ST-segment shift ≥ 0.1 mV from baseline at 80 milliseconds after the J point occurred in at least 2 contiguous leads (in the absence of Q waves). In the case of right bundlebranch block, the ST-segment shift was considered to be significant when it also occurred in leads V5 and V6. Electrocardiographic changes were not taken as criteria for positivity of the test in the absence of induced new wall motion abnormalities. However, the development of ST-segment depression ≥ 2 mm or ST-segment elevation ≥ 1.5 mm was considered to be significant enough for interruption of the test. In patients with LBBB, pre-existing ST-segment depression ≥ 0.1 mV or paced rhythm as well as in patients taking digitalis or antiarrhythmic medications, electrocardiographic changes were considered nondiagnostic.

### Statistical analysis

Values were expressed as the mean ± SD for continuous variables and as frequency and percentage for categorical variables. All data were analyzed by using SPSS 15.0 statistical software for Windows.

## Results

### Dobutamine doses and times of infusion (Table 2)

**Table 2:** Hemodynamic data of patients during stress echocardiography

*Hemodynamic and stress test variables*	*mean *± *SD*
Heart rate (beats/min)	
Rest	69.9 ± 12.1
Peak stress	123.1 ± 22.1
Systolic blood pressure (mm Hg)	
Rest	127.6 ± 18.1
Peak stress	136.3 ± 24.5
Rate-pressure product/100 (mm Hg/min)	
Rest	89.9 ± 22.8
Peak stress	167.6 ± 45.0
Dobutamine cumulative dose (μg/kg)	330 ± 105.2
Stress time (min)	6.6 ± 2.1
Target heart rate (%)	169 (63.8%)
Heart rate acceleration (beats/min^2^)	9.4 ± 5.9

Values are expressed as mean ± SD.

The mean weight-adjusted cumulative dose of dobutamine used was 330 ± 105.24 μg/kg. Total duration of dobutamine infusion with ADSE was 6.6 ± 2.1 min.

### Hemodynamic response (Table 2)

Heart rate rose from 69.9 ± 12.1 to 123.1 ± 22.1 beats/min at peak with a concomitant change in systolic blood pressure (127.6 ± 18.1 vs. 167.6 ± 45.0 mmHg).

Dobutamine administration produced a rapid increase in heart rate (9.4 ± 5.9 beats/min^2^).

Target heart rate was achieved in 169 out of 265 (63.8%) patients. Fifty three out of 96 (55%) patients without suboptimal chronotropic response were on therapy.

### Pharmacologic stress echocardiography and electrocardiographic results

Wall motion abnormalities were present at baseline in 114 (43%) patients. The test resulted positive in 61 patients because of the occurrence of new or worsening wall motion abnormalities. Ischemic electrocardiographic changes occurred in 68 (25.7%) patients.

### β-block

Only in 35 patients (13%) the administration of β-blockers at the end of the test was necessary.

### Safety and feasibility

No major side effects were observed during the test. Minor side effects are listed in Table 3.

**Table 3:** Adverse effects observed during accelerated dobutamine stress echocardiography

*Adverse effects*	*n*	*%*
*Symptoms*	13	4.9
Nausea	9	3.4
Symptomatic hypotension	3	1.1
Hypertension	0	0
Dyspnea	0	0
Headache	3	1.1
Anxiety	0	0
Tremor	1	0.4
General disconfort	1	0.4
*Arrhythmias*	64	24.2
Atrial fibrillation and flutter	2	0.75
PACs (six or more per minute)	4	1.5
Supraventricular tachycardia	1	0.4
Junctional rhythm	0	0
PVCs (six or more per minute)	57	21.5
NSVT	4	1.5
SVT	0	0
Second-degree AV block	1	0.4
Third-degree AV block	0	0
Bundle branch block.	3	1.1
Others	3	1.1

PACs, premature atrial complexes; PVCs, premature ventricular complexes; N/SVT, Non/sustained ventricular tachycardia.

Out of these effects the most frequent observed were nausea and PVCs.

Urination urgency was another side effect, never described before, frequently observed during the test (up to 30%). To obviate this problem, patients were kindly invited to urinate before beginning the test.

## Discussion

Continuous high dose dobutamine stress echocardography is a new protocol to add to those in use in all echocardographic laboratories. Other studies using an accelerated dobutamine infusion protocol have been reported [20-22]. In a previous study, Burger et al. using the same ADSE protocol, enrolled a smaller sample size of patients with suspected coronary artery disease and demonstrated that this protocol is safe, feasible and useful [1]. Our data confirm these results. Furthermore, our study showed that, in comparison with standard protocols, this new protocol required the administration of lower cumulative dobutamine dose. A significant reduction in test duration was also demonstrated [23-26]. Therefore, ADSE can be proposed as a useful test in busy laboratories with high volume of activity for the diagnostic imaging of myocardial ischemia in some subgroups of patients. However we recommend physicians to chose the most suitable test for each single patient.

In this preliminary experience, we demonstrated that the hemodynamic response is good and comparable with that found in our Echo-lab using the standard protocol.

This protocol did not consider atropine co-administration which represents the state of the art dosage regimen needed to optimise the diagnostic accuracy of the test. Nevertheless, target heart rate achievment was similar to that reported in large, multicenter studies using dobutamine and atropine [23-26].

The advantages of not employing atropine during stress were: reduced test time, reduced incidence of an unpleasant dry mouth, improved rate of performing diagnostic test in patients with narrow angle glaucoma, myasthenia gravis, obstructive uropathy or obstructive gastrointestinal disorders, reduced incidence of hypotension.

The ADSE protocol is a safe and well-tolerated method of performing stress echocardiography in patients with suspected or known coronary artery disease. Furthermore, side effects were similar to those described with the standard protocol [23-28].

### Limits

Uncommon side effects occurring during dobutamine stress echocardiography protocol need a larger sample size and a multicenter experience to be better evaluated, as demonstrated by EPIC- EDIC studies [27,29].

Our study represents the experience of a single centre, although a high flow and specialised one which cooperates with other institutional institutions in performing EPIC and EDIC studies and meets quality control requirements for stress echocardiographic readings.

This protocol is useful to evaluate myocardial ischemia although in the first 2 minutes it would be possible to study myocardial viability, since the dose of dobutamine corresponds approximately to the 10–20 μg/kg/min of the standard protocol.

The diagnostic accuracy and the prognostic power of ADSE was not assessed in this preliminary work. In the near future we shall evaluate these endpoints using coronary angiography for the anatomical assessment of coronary artery disease and myocardial scintigraphy, for the functional evaluation of inducible ischemia and long term clinical follow up data for the study of cardiac events.

## Conclusion

The ADSE is a new protocol using a continuous single high dose of dobutamine. Such protocol is a safe and well tolerated method of studying patients with known or suspected CAD that, using a low cumulative dobutamine dose, achieves target heart rate in short time, without co-administration of atropine.

Further investigation will be necessary to confirm our results and determine diagnostic accuracy and prognostic power of the test.

## Competing interests

The author(s) declare that they have no competing interests.

## Authors' contributions

GM made substantial contributions to conception and design of the study; GM, CM, GP and PGP made substantial contributions in acquisition of clinical and echocardiographic data; GM, HP and MS have been involved in drafting the manuscript; HP and MS performed the statistical analysis; GM has given final approval of the version to be published. All authors read and approved the final manuscript.
